# A new hydrated crystalline form of *N*-[(*E*)-(4-hy­droxy­phen­yl)methyl­idene]-1*H*-1,2,4-triazol-3-amine and its anti­fungal activity

**DOI:** 10.1107/S205698902401209X

**Published:** 2025-01-01

**Authors:** Boualia Boutheina, Bouhidel Zakaria, Aouatef Cherouana, Bendeif El-Eulmi

**Affiliations:** a Unité de Recherche de Chimie de l'Environnement et Moléculaire Structurale (URCHEMS) Département de Chimie Université Mentouri de Constantine 25000 Constantine Algeria; b Synchrotron SOLEIL, L’Orme des Merisiers, BP48, Saint Aubin, 91192, Gif-sur-Yvette, France; c Laboratoire de Cristallographie, Résonance Magnétique et Modélisation, CRM2 UMR 7036 Institut Jean Barriol Faculté des Sciences et Technologies BP 70239 54506 Vandoeuvre lès Nancy France; Venezuelan Institute of Scientific Research, Venezuela

**Keywords:** Schiff bases, single-crystal, X-ray diffraction, hydrogen bonding, inter­molecular inter­actions, Hirshfeld surface analysis, anti­fungal activity

## Abstract

The synthesis, crystal structure, Hirshfeld analysis, and anti­fungal assessment of a new monohydrated Schiff base with a triazole moiety are reported. The structural study revealed the presence of three significant hydrogen bonds (N—H⋯N, O—H⋯N, and O—H⋯O), which contribute to the cohesion of the crystal. These bonds generate two-dimensional layers parallel to the *bc* plane, built on the basis of rings with the graph-set motifs *R*^4^_4_(8) and *R*^4^_4_(24). The crystal structure is further consolidated by π–π inter­actions between similar rings. The anti­fungal activity of the Schiff base was evaluated against three fungi: *Fusarium oxysporum*, *Botrytis cinerea*, and *Alternaria alternata*, showing significant anti­fungal activity, particularly against *Alternaria alternata*.

## Abstract 2

The synthesis, crystal structure, Hirshfeld surface analysis, and anti­fungal assessment of a new monohydrated Schiff base with a triazole moiety are reported.

## 1. Chemical context

Plant fungal diseases represent a major obstacle to agricultural development, leading to substantial economic losses. Chemical fungicides remain widely used as effective and affordable solutions for the prevention and control of these diseases. Research is currently focused on developing new pesticide mol­ecules with broad biological activity, high efficacy, and low toxicity (Bai *et al.*, 2019 ▸).

Our team aims to synthesize new mol­ecules with promising applications, particularly in the biological field, such as anti­microbial and anti­fungal agents. To this end, various aromatic Schiff bases have been previously studied and reported (Moussa Slimane *et al.*, 2022 ▸; Benarous *et al.*, 2022 ▸; Maza *et al.*, 2020 ▸; Bouhidel *et al.*, 2018 ▸). This family of compounds contains an imine functional group (–C=N–) formed by the condensation of primary amines and carbonyl compounds. They are of great inter­est due to their diverse synthetic and biological applications (Kirubavathy *et al.*, 2017 ▸). These compounds can exist in two tautomeric forms, enol and ketone, due to intra­molecular proton transfer, and their C=N bond is crucial for various biological activities, including anti­bacterial and anti­fungal properties (Wu *et al.*, 2019 ▸; PrabhuKumar *et al.*, 2022 ▸; Kumar *et al.*, 2023 ▸). Studies on the characteristics of these compounds affected by tautomerism, mol­ecular geometry, and crystal structure have led to the synthesis and investigation of numerous Schiff bases.

In this paper, we present the synthesis, structural characterization, Hirshfeld surface analysis, and anti­fungal properties of a new Schiff base, *N*-[(*E*)-(4-hy­droxy­phen­yl)methyl­idene]-1*H*-1,2,4-triazol-3-amine, which was obtained through a one-step reflux reaction (see the *Synthesis and crystallization* section).
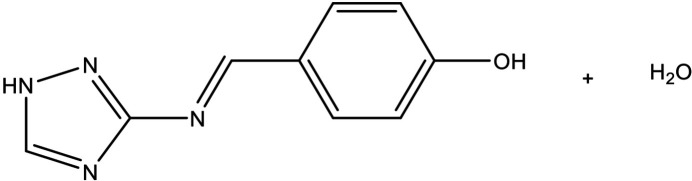


## 2. Structural commentary

**Figure 1:**
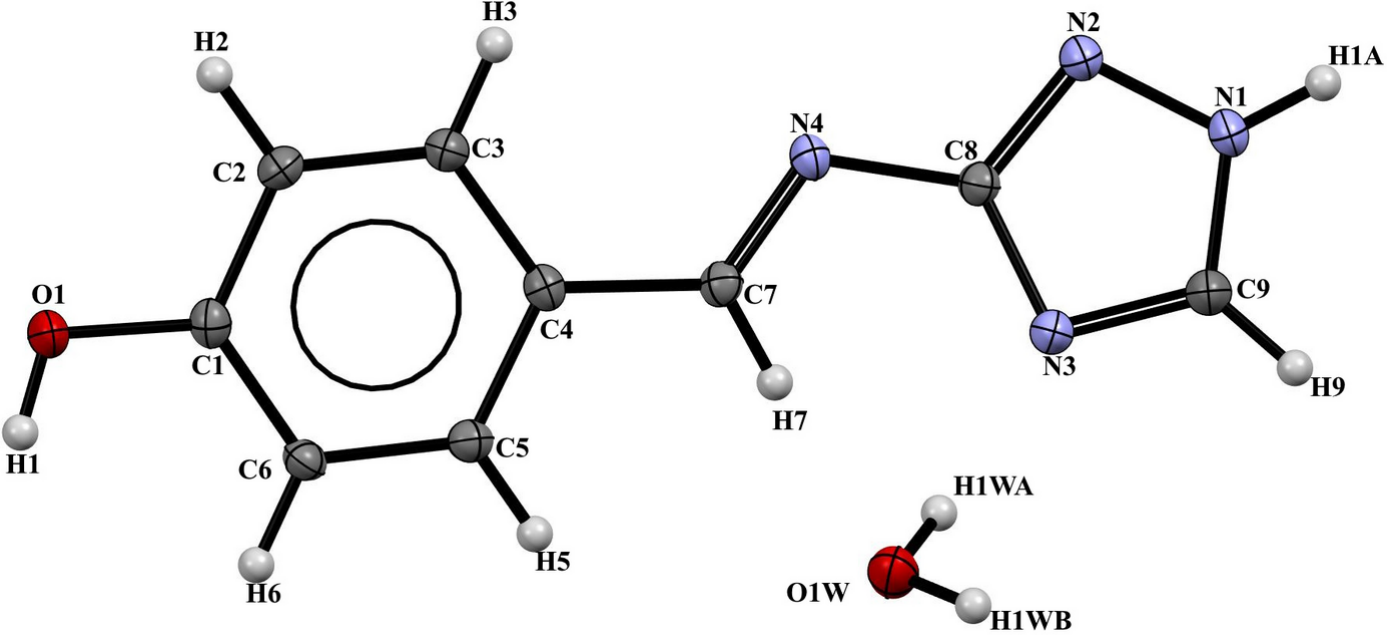
View of the title compound with the atom-numbering scheme. Displacement ellipsoids for non-H atoms are drawn at the 50% probability level.

The crystal structure of the monohydrated title compound (L1) is based on two aromatic rings connected by an azomethine group. These rings consist of a benzene ring and a 1,2,4-triazole ring, with the benzene ring mono-para-substituted by a hydroxyl group (Fig. 1 ▸). The Schiff base adopts an *(E)* conformation relative to the N4=C7 imine bond, displaying a torsion angle of 172.5 (2)°. Bond lengths and angles (Table 1 ▸) are consistent with those observed in previously reported similar structures (Maza *et al.*, 2020 ▸; Kołodziej *et al.*, 2019 ▸; Bouhidel *et al.*, 2018 ▸). The mol­ecule is relatively planar, with a dihedral angle of 17.68 (8)° between the two aromatic rings.

## 3. Supra­molecular features

**Figure 2:**
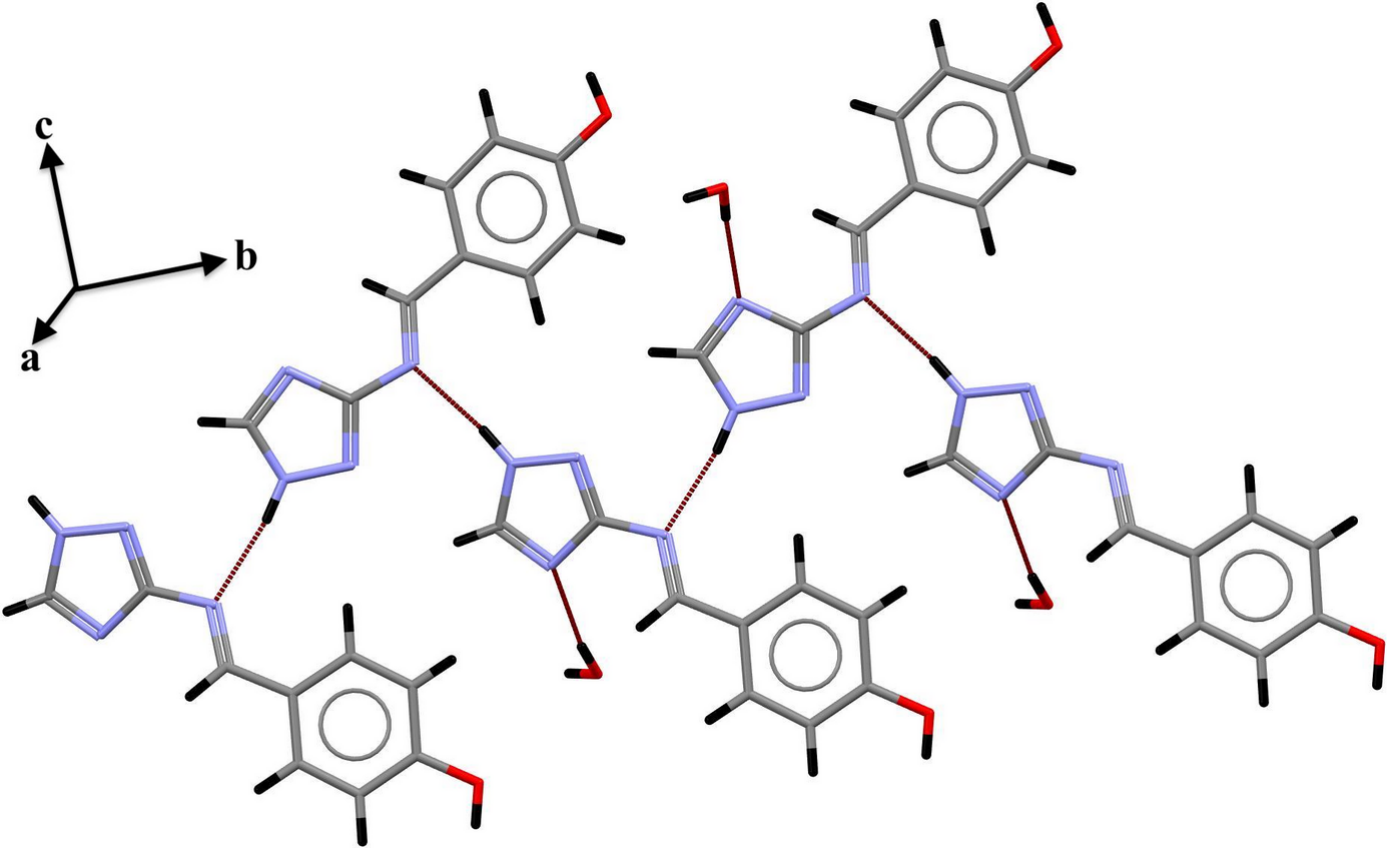
Chains of N—H⋯N hydrogen bonds in the title compound.

**Figure 3:**
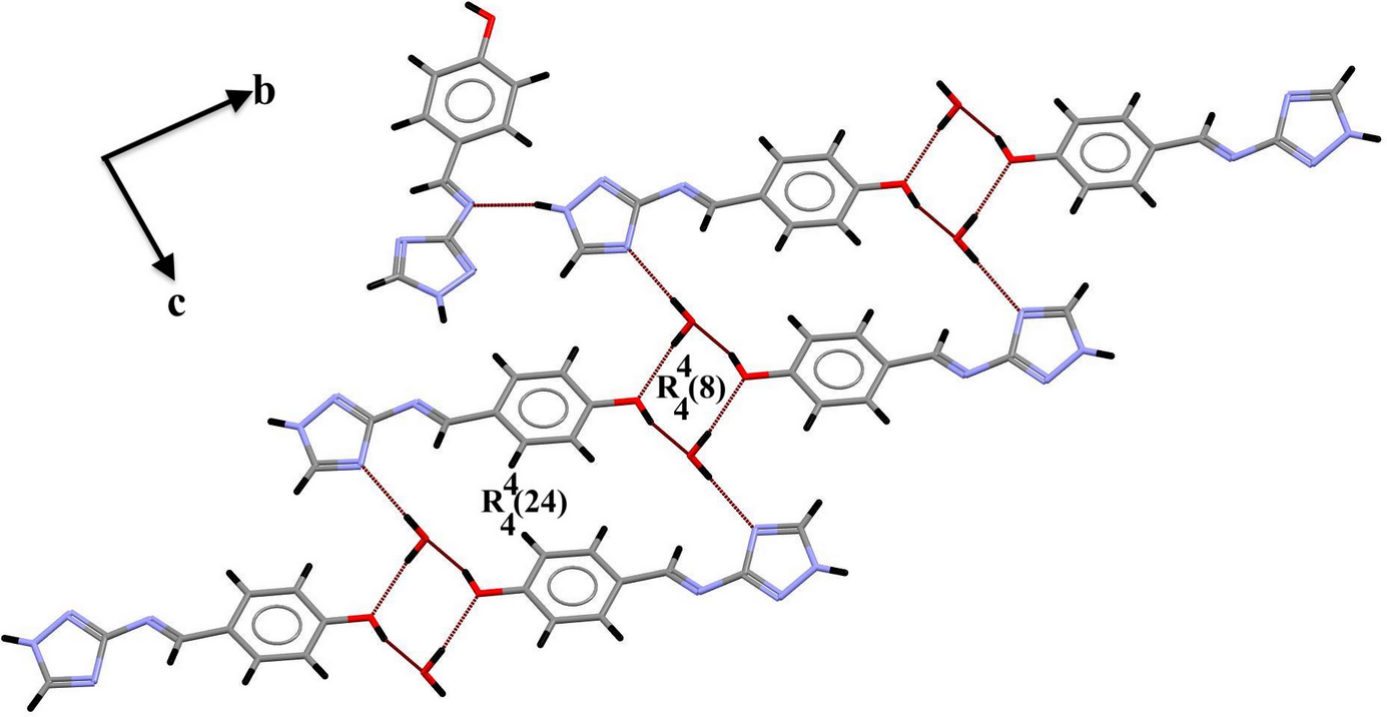
Two-dimensional rings formed by the combination of the three types of hydrogen bonds.

**Figure 4:**
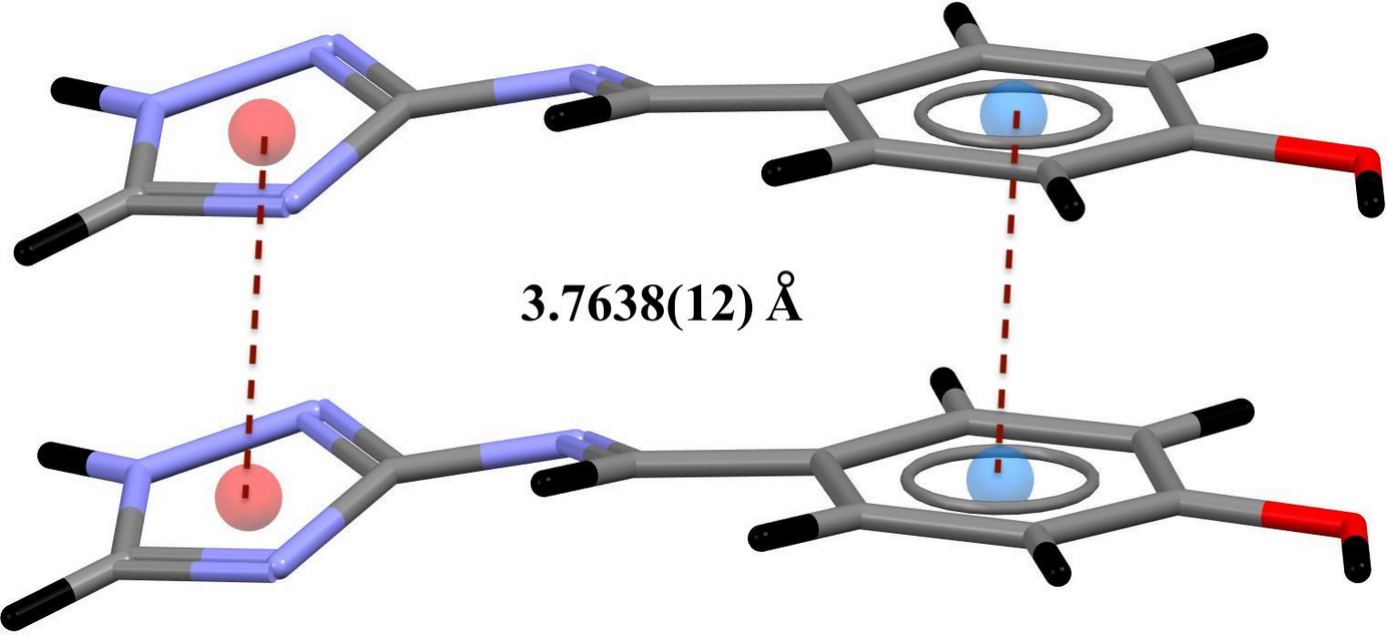
π**–**π inter­actions between similar rings.

The crystal structure of (L1) is consolidated by N—H⋯N, O—H⋯O, and O—H⋯N hydrogen bonds (Table 2 ▸). The N—H⋯N hydrogen bond forms between the nitro­gen atom of the triazole (N1) and the azomethine nitro­gen (N4), creating infinite chains that extend along the *b-*axis direction (Fig. 2 ▸). The combination of this hydrogen bond with those involving the water mol­ecule (O—H⋯O and O—H⋯N) generates two-dimensional layers parallel to the *bc* plane, based on rings with 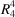
(8) and 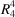
(24) graph-set motifs (Etter *et al.*, 1990 ▸; Bernstein *et al.*, 1995 ▸) (Fig. 3 ▸). The crystal structure is further consolidated by π–π inter­actions between similar rings (Fig. 4 ▸) with centroid–centroid distances of 3.7638 (15) Å.

## 4. Database survey

A search of the Cambridge Structural Database (CSD, version 5.45, March 2024 update; Groom *et al.*, 2016 ▸) using the Schiff base framework incorporating a 1,2,4-triazole yielded only four results. Among these, two compounds from our team’s work were identified, featuring a bromine substituent replacing the hydroxyl group: TIVDUA (Maza *et al.*, 2020 ▸). The remaining three results: PEVXAS (Brink *et al.*, 2018 ▸), TIVFAI (Kolodziej *et al.*, 2019 ▸), and UZOKIE (Chohan & Hanif, 2011 ▸) contain structures similar to the one reported in this article, with different substituents such as methyl, bromine, and/or hydroxyl. The compounds were characterized using a range of spectroscopic techniques, including FTIR, UV-Vis, and NMR, and their structures were determined by single-crystal X-ray diffraction. These four studies highlight the significance of Schiff bases as versatile compounds with a wide array of applications. The synthesis and characterization of novel Schiff bases derived from 3-amino-1*H*-1,2,4-triazole opens up new possibilities for the development of potential therapeutic agents.

## 5. Hirshfeld surface analysis

**Figure 5:**
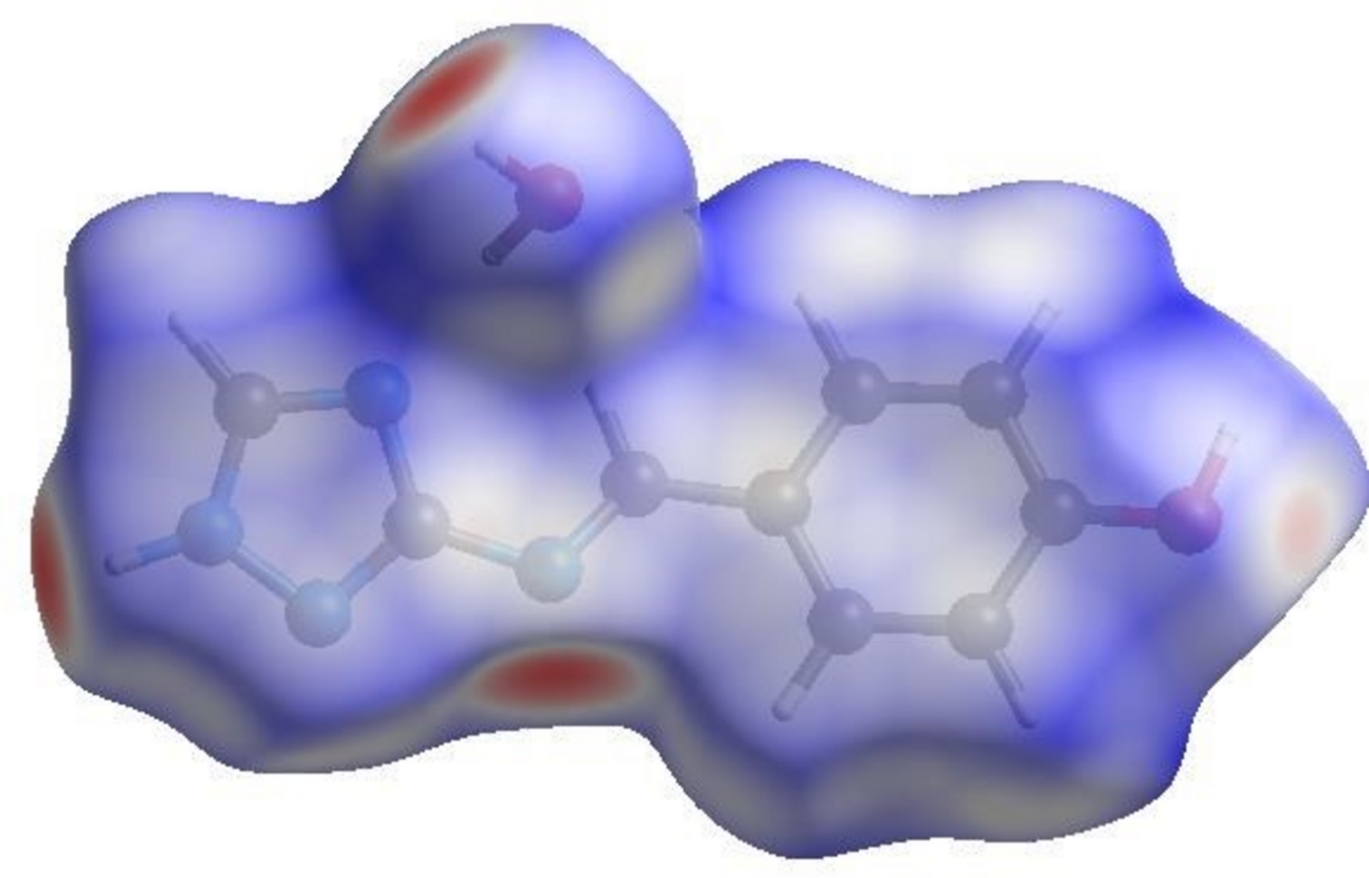
Hirshfeld surface (*d*_norm_) of the studied crystal.

**Figure 6:**
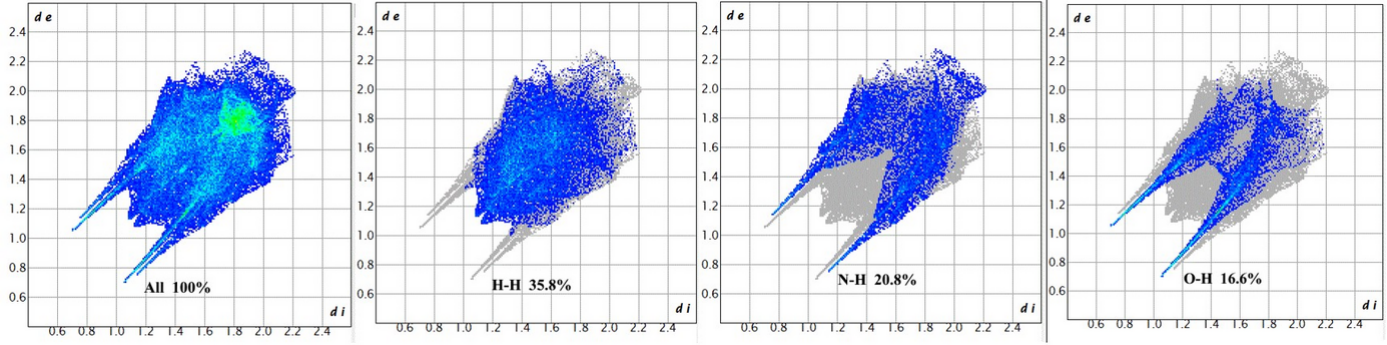
Two-dimensional fingerprint plots of the compound under study, showing H—H, H⋯N/N⋯H, and H⋯O/O⋯H contacts.

To further analyze the inter­molecular inter­actions, Hirshfeld surfaces (HS) were examined using graphical tools (Spackman & Jayatilaka, 2009 ▸; Spackman *et al.*, 2021 ▸). Fig. 5 ▸ illustrates the Hirshfeld surface of the compound, mapped over *d*_norm_, where the colors indicate different types of contacts: red areas represent shorter contacts, white areas denote contacts equal to the sum of van der Waals radii, and blue areas represent longer contacts. The compound exhibits a short inter­molecular H⋯H contact, comprising approximately 35.8% of the total inter­molecular inter­actions (Figs. 6 ▸ and 7 ▸). The 2D fingerprint plots reveal a notable contribution from H⋯N/N⋯H inter­actions, accounting for about 20.8%, shown by a pair of sharp peaks at around 1.8 Å. Moreover, O—H⋯O hydrogen bonds involving the water mol­ecules contribute 16.6% to the crystal structure inter­actions.

## 6. Synthesis and crystallization

All chemicals were commercially available, purchased from Sigma-Aldrich, and used as received without purification. To a solution of 4-hy­droxy­benzaldehyde (0.224 g, 2 mmol) in ethanol (15 mL), 3-amino-1*H*-1,2,4-triazole (0.168 g, 2 mmol) and a few drops of acetic acid were added. The reaction mixture was stirred under reflux at 373 K for 6 h. Following this, the whitish solution was cooled in an ice bath. The resulting crystalline powder was filtered, washed with ethanol, and dried under vacuum. Pure colorless crystals of (L1) were then obtained by recrystallization from a solvent mixture of aceto­nitrile and water.

## 7. Refinement

Crystal data, data collection and structure refinement details are summarized in Table 3 ▸. All H atoms were located in difference electron-density maps and were treated as riding on their parent atoms.

## 8. Anti­fungal activity

Triazole rings are well-known for their effectiveness against many phytopathogenic fungi (Colley *et al.*, 2019 ▸; Herbrecht, 2004 ▸). In this work, the anti­fungal activity of the compound against three fungal strains is reported: *Fusarium oxysporum*, *Botrytis cinerea*, and *Alternaria alternata*. These fungi are known to cause various plant diseases. Standard anti­biotics were used as positive controls (Carbendazim for *Fusarium oxysporum*, and Thia­bendazole for both *Botrytis cinerea* and *Alternaria alternata*).

The evaluation of anti­fungal activity was conducted using the agar diffusion method, specifically the disc diffusion method, cultured in Potato Dextrose Agar (PDA) medium, with various concentrations of the compounds in 90 mm diameter Petri dishes. PDA was also used as a culture medium for the isolation, purification of strains, and for obtaining the inoculum, as it promotes rapid growth and abundant sporulation.

This study was performed *in vitro*, utilizing mycelial growth tests from young cultures aged one week on solid PDA medium (final volume of 20 ml). The tested compound was dissolved in DMSO to prepare three concentrations: 12.5, 25, and 50 µg ml^−1^. The tests were conducted in quadruplicate. Mycelial plugs (6 mm in diameter) were taken from the margins of the actively growing mycelium in each culture and placed in the center of Petri dishes containing PDA medium amended with the triazole-based Schiff bases. Isolates of the three fungi were tested with a range of concentrations of the studied compound. The growth of each colony was measured along two perpendicular diameters, and the average radius of each colony was calculated by subtracting the radius of the initial inoculum disk. The percentage inhibition was then calculated using the following formula (Zhang *et al.*, 2019 ▸).

**Inhibition rate % = (diameter of control mycelium - diameter of treated mycelium)/(diameter of control mycelium x 100)**

**Figure 8:**
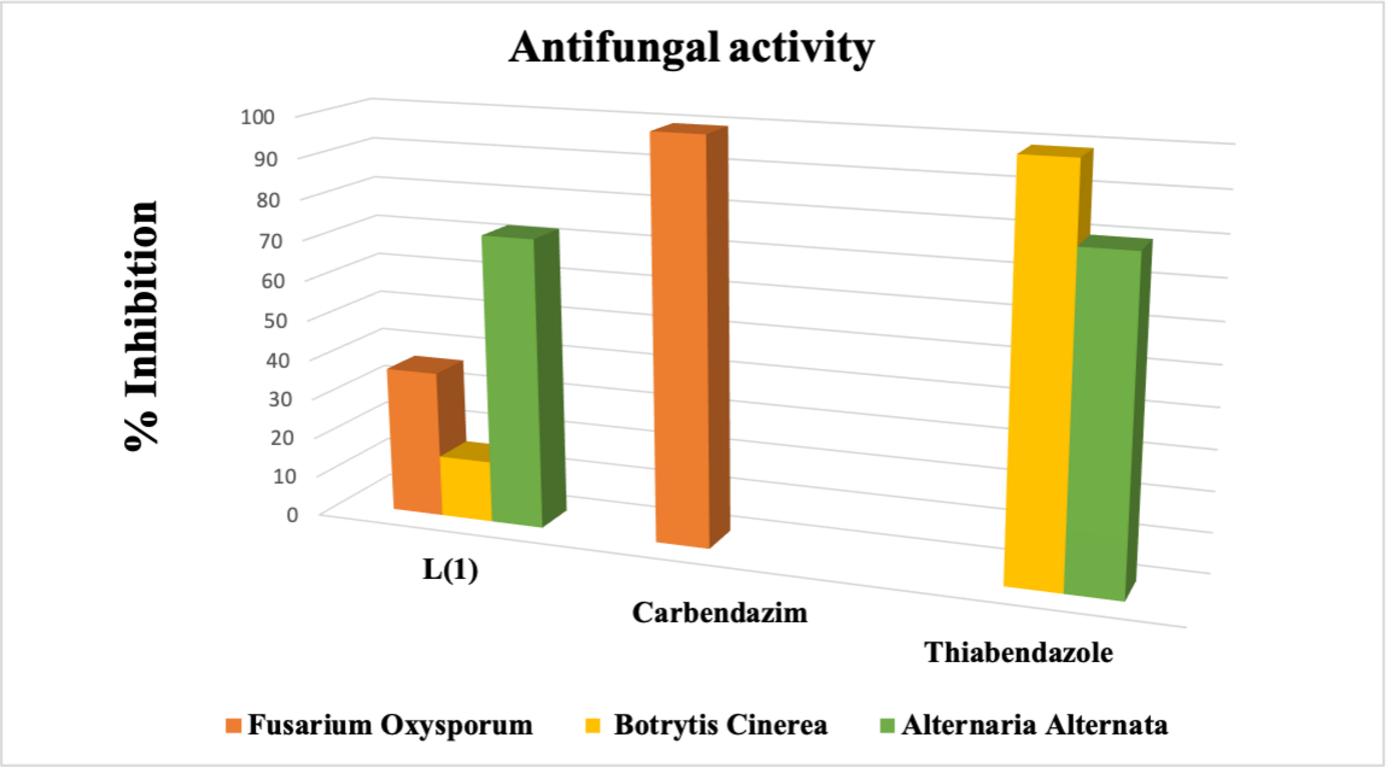
Histogram showing the percentage of inhibition of the ligand against the phytopathogens *Fusarium Oxysporum*, *Botrytis Cinerea* and *Alternaria Alternata*.

The best results obtained with the title compound were at a concentration of 12.5 µg ml^−1^, as reported in Table 4 ▸ and Fig. 8 ▸.

The results of the inhibition activity assessment indicate that all three tested fungi are sensitive to the anti­fungal action of this Schiff base. Variations in inhibition were observed among the different fungal strains. The tested compound exhibits broad-spectrum activity, significantly inhibiting the mycelial growth of *Alternaria alternata*, with an inhibition percentage of 72.28%, which is very close to that of the positive control (79.8%).

For the other two fungi, *Botrytis cinerea* and *Fusarium oxysporum*, the most pronounced anti­fungal effect was observed with *Fusarium oxysporum*, which demonstrated an inhibition percentage of 36%. In contrast, *Botrytis cinerea* exhibited a lower inhibition percentage of 15.33%. The inhibition percentages obtained for these fungi are slightly lower than those of the positive control.

## Supplementary Material

Crystal structure: contains datablock(s) global, I. DOI: 10.1107/S205698902401209X/zn2040sup1.cif

Structure factors: contains datablock(s) I. DOI: 10.1107/S205698902401209X/zn2040Isup2.hkl

Supporting information file. DOI: 10.1107/S205698902401209X/zn2040Isup3.cml

CCDC reference: 2409810

Additional supporting information:  crystallographic information; 3D view; checkCIF report

## Figures and Tables

**Figure 7:**
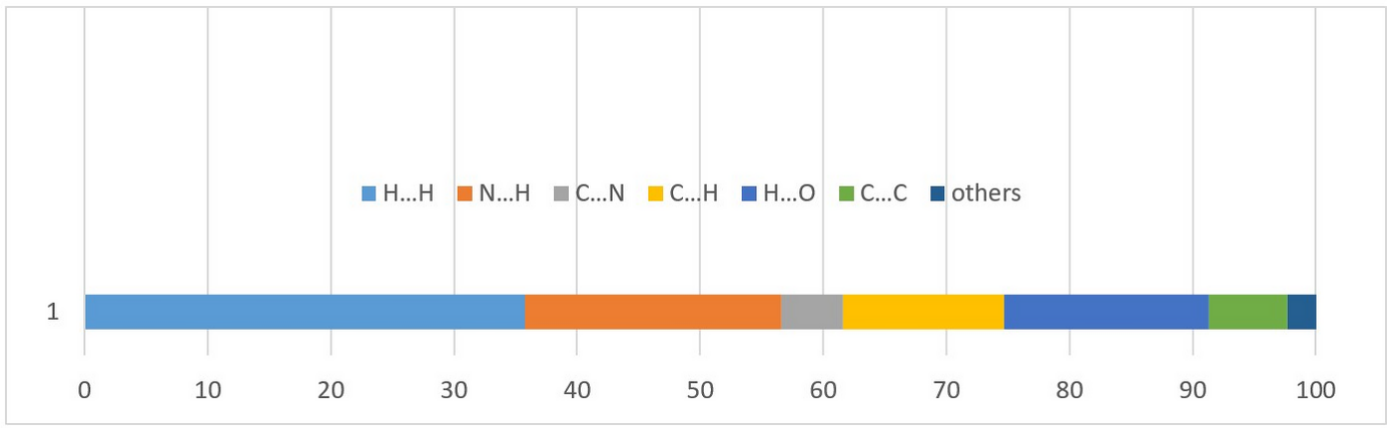
Proportional contributions of different inter­actions to the Hirshfeld surface of the title compound.

**Table 1:** Selected geometric parameters (Å, °)

O1—C1	1.3629 (19)	N3—C8	1.371 (2)
N1—N2	1.3655 (18)	N3—C9	1.330 (2)
N1—C9	1.331 (2)	N4—C7	1.293 (2)
N2—C8	1.335 (2)	N4—C8	1.405 (2)
			
N2—N1—C9	110.23 (12)	N4—C7—C4	124.95 (13)
N1—N2—C8	101.81 (12)	N2—C8—N3	114.88 (13)
C8—N3—C9	102.04 (13)	N2—C8—N4	120.10 (13)
C7—N4—C8	116.10 (12)	N3—C8—N4	124.92 (14)
O1—C1—C6	117.37 (13)	N1—C9—N3	111.04 (14)
O1—C1—C2	122.07 (14)		

**Table 2:** Hydrogen-bond geometry (Å, °)

*D*—H⋯*A*	*D*—H	H⋯*A*	*D*⋯*A*	*D*—H⋯*A*
O1—H1⋯O1*W*^i^	0.84	1.89	2.7060 (17)	163
O1*W*—H1*WA*⋯N3	0.87	2.06	2.9147 (19)	166
N1—H1*A*⋯N4^ii^	0.88	2.03	2.895 (2)	168
O1*W*—H1*WB*⋯O1^iii^	0.87	1.98	2.8217 (18)	161
C7—H7⋯N3	0.95	2.41	2.785 (2)	103

Symmetry codes: (i) 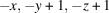; (ii) 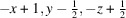; (iii) 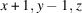.

**Table 3:** Experimental details

Crystal data	
Chemical formula	C_9_H_10_N_4_O_2_
*M*_r_	206.21
Crystal system, space group	Monoclinic, *P*2_1_/*c*
Temperature (K)	100
*a*, *b*, *c* (Å)	3.7638 (1), 9.286 (3), 26.194 (2)
β (°)	93.786 (2)
*V* (Å^3^)	913.5 (3)
*Z*	4
Radiation type	Mo *K*α
μ (mm^−1^)	0.11
Crystal size (mm)	0.10 × 0.10 × 0.09

Data collection	
Diffractometer	Nonius KappaCCD
No. of measured, independent and observed [*I* > 2σ(*I*)] reflections	11656, 2769, 1890
*R*_int_	0.052
(sin θ/λ)_max_ (Å^−1^)	0.713

Refinement	
*R*[*F*^2^ > 2σ(*F*^2^)], *wR*(*F*^2^), *S*	0.048, 0.139, 1.10
No. of reflections	2769
No. of parameters	139
H-atom treatment	H-atom parameters constrained
Δρ_max_, Δρ_min_ (e Å^−3^)	0.36, −0.28

Computer programs: *CrysAlis CCD* and *CrysAlis RED* (Rigaku OD, 2017 ▸), *SHELXL* (Sheldrick, 2015 ▸a), *SHELXL* (Sheldrick, 2015 ▸b) and *OLEX2* (Dolomanov *et al.*, 2009 ▸).

**Table 4:** Percentage of inhibition of the title compound against *Fusarium oxysporum*, *Botrytis cinerea* and *Alternaria alternata*

	*Fusarium oxysporum*	*Botrytis cinerea*	*Alternaria alternata*
12.5 µg ml^−1^ (L1)	36.62±0.70	15.33±0.50	72.28±1.25
Carbendazim	99.6±0.10	–	–
Thia­bendazole	–	99.1±1.30	79.8±0.45

## supplementary crystallographic information

### N-[(E)-(4-Hydroxyphenyl)methylidene]-1H-1,2,4-triazol-3-amine Crystal data

**Table:**

C_9_H_10_N_4_O_2_	*F*(000) = 432
*M**_r_* = 206.21	*D*_x_ = 1.499 Mg m^−^^3^
Monoclinic, *P*2_1_/*c*	Mo *K*α radiation, λ = 0.71073 Å
Hall symbol: -P 2ybc	Cell parameters from 11656 reflections
*a* = 3.7638 (1) Å	θ = 2.7–30.5°
*b* = 9.286 (3) Å	µ = 0.11 mm^−^^1^
*c* = 26.194 (2) Å	*T* = 100 K
β = 93.786 (2)°	Prism, colourless
*V* = 913.5 (3) Å^3^	0.10 × 0.10 × 0.09 mm
*Z* = 4	

### N-[(E)-(4-Hydroxyphenyl)methylidene]-1H-1,2,4-triazol-3-amine Data collection

**Table:**

Nonius KappaCCD diffractometer	1890 reflections with *I* > 2σ(*I*)
Radiation source: fine-focus sealed tube	*R*_int_ = 0.052
Graphite monochromator	θ_max_ = 30.5°, θ_min_ = 2.7°
Detector resolution: 18.4 pixels mm^-1^	*h* = −5→3
ω scans	*k* = −13→13
11656 measured reflections	*l* = −37→36
2769 independent reflections	

### N-[(E)-(4-Hydroxyphenyl)methylidene]-1H-1,2,4-triazol-3-amine Refinement

**Table:**

Refinement on *F*^2^	0 restraints
Least-squares matrix: full	Hydrogen site location: mixed
*R*[*F*^2^ > 2σ(*F*^2^)] = 0.048	H-atom parameters constrained
*wR*(*F*^2^) = 0.139	W = 1/[Σ^2^(*F*O^2^) + (0.0656*P*)^2^ + 0.0206*P*] WHERE *P* = (*F*O^2^ + 2*F*C^2^)/3
*S* = 1.10	(Δ/σ)_max_ < 0.001
2769 reflections	Δρ_max_ = 0.36 e Å^−^^3^
139 parameters	Δρ_min_ = −0.28 e Å^−^^3^

### N-[(E)-(4-Hydroxyphenyl)methylidene]-1H-1,2,4-triazol-3-amine Special details

**Table:**

Geometry. Bond distances, angles etc. have been calculated using the rounded fractional coordinates. All su's are estimated from the variances of the (full) variance-covariance matrix. The cell esds are taken into account in the estimation of distances, angles and torsion angles

### N-[(E)-(4-Hydroxyphenyl)methylidene]-1H-1,2,4-triazol-3-amine Fractional atomic coordinates and isotropic or equivalent isotropic displacement parameters (Å^2^)

**Table:**

	*x*	*y*	*z*	*U*_iso_*/*U*_eq_	
O1	−0.1647 (3)	0.88452 (11)	0.47038 (4)	0.0185 (3)	
N1	0.4773 (3)	0.06341 (13)	0.25638 (5)	0.0163 (3)	
N2	0.4122 (3)	0.20806 (13)	0.25489 (5)	0.0162 (3)	
N3	0.3004 (3)	0.11316 (13)	0.33251 (5)	0.0171 (4)	
N4	0.2200 (3)	0.37277 (13)	0.31666 (5)	0.0143 (3)	
C1	−0.1362 (4)	0.76242 (16)	0.44207 (6)	0.0141 (4)	
O1W	0.4942 (3)	0.14542 (12)	0.44149 (4)	0.0217 (3)	
C2	−0.2342 (4)	0.62794 (15)	0.45996 (6)	0.0140 (4)	
C3	−0.1841 (4)	0.50761 (16)	0.43005 (5)	0.0143 (4)	
C4	−0.0353 (4)	0.51821 (16)	0.38247 (6)	0.0129 (4)	
C5	0.0497 (4)	0.65532 (16)	0.36412 (6)	0.0144 (4)	
C6	−0.0017 (4)	0.77665 (16)	0.39366 (6)	0.0149 (4)	
C7	0.0418 (4)	0.38374 (15)	0.35701 (6)	0.0135 (4)	
C8	0.3027 (4)	0.23219 (16)	0.30156 (6)	0.0136 (4)	
C9	0.4127 (4)	0.01051 (17)	0.30206 (6)	0.0178 (4)	
H1	−0.23441	0.86312	0.49923	0.0280*	
H1A	0.55125	0.01248	0.23081	0.0200*	
H2	−0.33393	0.61887	0.49219	0.0170*	
H3	−0.25199	0.41572	0.44203	0.0170*	
H5	0.14259	0.66495	0.33143	0.0170*	
H6	0.05423	0.86934	0.38109	0.0180*	
H7	−0.04532	0.29740	0.37110	0.0160*	
H9	0.44289	−0.08761	0.31165	0.0210*	
H1WA	0.40240	0.13060	0.41063	0.0330*	
H1WB	0.64128	0.07412	0.44732	0.0330*	

### N-[(E)-(4-Hydroxyphenyl)methylidene]-1H-1,2,4-triazol-3-amine Atomic displacement parameters (Å^2^)

**Table:**

	*U*^11^	*U*^22^	*U*^33^	*U*^12^	*U*^13^	*U*^23^
O1	0.0281 (6)	0.0130 (5)	0.0149 (6)	0.0007 (4)	0.0050 (5)	−0.0020 (4)
N1	0.0209 (6)	0.0137 (6)	0.0148 (6)	0.0010 (5)	0.0050 (5)	−0.0028 (5)
N2	0.0209 (6)	0.0135 (6)	0.0146 (6)	−0.0006 (5)	0.0043 (5)	−0.0012 (5)
N3	0.0241 (6)	0.0129 (6)	0.0149 (7)	0.0003 (5)	0.0050 (5)	0.0000 (5)
N4	0.0170 (6)	0.0129 (6)	0.0132 (6)	0.0005 (5)	0.0015 (5)	−0.0023 (5)
C1	0.0138 (6)	0.0135 (7)	0.0148 (7)	0.0016 (5)	−0.0006 (5)	−0.0020 (6)
O1W	0.0299 (6)	0.0187 (6)	0.0164 (6)	0.0037 (5)	0.0005 (5)	0.0000 (4)
C2	0.0145 (6)	0.0158 (7)	0.0120 (7)	0.0017 (5)	0.0035 (5)	0.0005 (5)
C3	0.0146 (6)	0.0145 (7)	0.0137 (7)	−0.0001 (6)	0.0014 (5)	0.0017 (5)
C4	0.0123 (6)	0.0138 (7)	0.0127 (7)	0.0005 (5)	0.0007 (5)	−0.0019 (5)
C5	0.0164 (7)	0.0138 (7)	0.0132 (7)	0.0006 (5)	0.0027 (6)	0.0006 (5)
C6	0.0173 (7)	0.0119 (7)	0.0157 (7)	−0.0008 (5)	0.0022 (6)	0.0015 (6)
C7	0.0135 (6)	0.0130 (7)	0.0137 (7)	−0.0018 (5)	−0.0002 (5)	−0.0008 (5)
C8	0.0149 (6)	0.0129 (7)	0.0130 (7)	−0.0014 (5)	0.0012 (5)	−0.0008 (5)
C9	0.0238 (7)	0.0144 (7)	0.0158 (8)	0.0012 (6)	0.0049 (6)	0.0006 (6)

### N-[(E)-(4-Hydroxyphenyl)methylidene]-1H-1,2,4-triazol-3-amine Geometric parameters (Å, º)

**Table:**

O1—C1	1.3629 (19)	C3—C4	1.403 (2)
N1—N2	1.3655 (18)	C4—C5	1.405 (2)
N1—C9	1.331 (2)	C4—C7	1.454 (2)
O1—H1	0.8400	C5—C6	1.388 (2)
N2—C8	1.335 (2)	O1W—H1WB	0.8700
N3—C8	1.371 (2)	O1W—H1WA	0.8700
N3—C9	1.330 (2)	C2—H2	0.9500
N4—C7	1.293 (2)	C3—H3	0.9500
N4—C8	1.405 (2)	C5—H5	0.9500
C1—C6	1.402 (2)	C6—H6	0.9500
N1—H1A	0.8800	C7—H7	0.9500
C1—C2	1.392 (2)	C9—H9	0.9500
C2—C3	1.385 (2)		
			
N2—N1—C9	110.23 (12)	N2—C8—N3	114.88 (13)
C1—O1—H1	109.00	N2—C8—N4	120.10 (13)
N1—N2—C8	101.81 (12)	N3—C8—N4	124.92 (14)
C8—N3—C9	102.04 (13)	N1—C9—N3	111.04 (14)
C7—N4—C8	116.10 (12)	H1WA—O1W—H1WB	105.00
O1—C1—C6	117.37 (13)	C1—C2—H2	121.00
C2—C1—C6	120.56 (14)	C3—C2—H2	121.00
C9—N1—H1A	125.00	C4—C3—H3	119.00
O1—C1—C2	122.07 (14)	C2—C3—H3	119.00
N2—N1—H1A	125.00	C4—C5—H5	120.00
C1—C2—C3	118.97 (14)	C6—C5—H5	120.00
C2—C3—C4	121.55 (14)	C5—C6—H6	120.00
C3—C4—C5	118.73 (14)	C1—C6—H6	120.00
C3—C4—C7	116.79 (13)	N4—C7—H7	118.00
C5—C4—C7	124.36 (14)	C4—C7—H7	118.00
C4—C5—C6	120.11 (14)	N1—C9—H9	124.00
C1—C6—C5	119.98 (14)	N3—C9—H9	124.00
N4—C7—C4	124.95 (13)		
			
C9—N1—N2—C8	−1.03 (15)	C6—C1—C2—C3	2.5 (2)
N2—N1—C9—N3	0.71 (17)	O1—C1—C6—C5	176.87 (14)
N1—N2—C8—N3	1.06 (16)	C2—C1—C6—C5	−2.9 (2)
N1—N2—C8—N4	177.48 (12)	C1—C2—C3—C4	0.4 (2)
C9—N3—C8—N2	−0.67 (17)	C2—C3—C4—C5	−2.8 (2)
C9—N3—C8—N4	−176.90 (14)	C2—C3—C4—C7	173.30 (14)
C8—N3—C9—N1	−0.05 (16)	C3—C4—C5—C6	2.3 (2)
C8—N4—C7—C4	172.52 (14)	C7—C4—C5—C6	−173.45 (15)
C7—N4—C8—N2	164.83 (14)	C3—C4—C7—N4	−170.48 (15)
C7—N4—C8—N3	−19.1 (2)	C5—C4—C7—N4	5.4 (2)
O1—C1—C2—C3	−177.31 (14)	C4—C5—C6—C1	0.5 (2)

### N-[(E)-(4-Hydroxyphenyl)methylidene]-1H-1,2,4-triazol-3-amine Hydrogen-bond geometry (Å, º)

**Table:**

*D*—H···*A*	*D*—H	H···*A*	*D*···*A*	*D*—H···*A*
O1—H1···O1*W*^i^	0.84	1.89	2.7060 (17)	163
O1*W*—H1*WA*···N3	0.87	2.06	2.9147 (19)	166
N1—H1*A*···N4^ii^	0.88	2.03	2.895 (2)	168
O1*W*—H1*WB*···O1^iii^	0.87	1.98	2.8217 (18)	161
C7—H7···N3	0.95	2.41	2.785 (2)	103

Symmetry codes: (i) −*x*, −*y*+1, −*z*+1; (ii) −*x*+1, *y*−1/2, −*z*+1/2; (iii) *x*+1, *y*−1, *z*.
